# Effect of reduced orbital rotation on image quality and intra-articular screw detection in intraoperative 3D imaging of proximal humerus plate fixation: a cadaveric study

**DOI:** 10.1186/s13018-026-06800-9

**Published:** 2026-04-05

**Authors:** Benno Bullert, Livia Morlock, Fenna Brunken, Paul A. Gruetzner, Sven Y. Vetter, Nils Beisemann

**Affiliations:** Research group Medical Imaging and Navigation in Trauma and Orthopaedic Surgery (MINTOS), Department for Orthopaedics and Trauma Surgery, BG Klinik Ludwigshafen, Heidelberg University, Ludwig-Guttmann-Str. 13, 67071 Ludwigshafen, Germany

**Keywords:** Proximal humerus fracture, PHILOS plate osteosynthesis, Intraoperative 3D imaging, Tomosynthesis, Metal artifact reduction (MAR), Screw perforation detection

## Abstract

**Background:**

Proximal humerus fractures are common injuries in the elderly, frequently treated with plate osteosynthesis. Intraoperative 2D fluoroscopy provides only limited visualization of the humeral head’s complex anatomy, often leading to undetected intra-articular screw perforations. Intraoperative 3D imaging provides enhanced assessment but is technically limited by the confined operating field and the risk of collisions between the C-arm, patient, and operating table. This cadaveric study systematically investigated how progressive reduction of orbital rotation during intraoperative 3D imaging of the proximal humerus affects image quality and intra-articular screw detection accuracy in plate osteosynthesis.

**Methods:**

Five fresh-frozen cadaveric shoulders were scanned using an isocentric C-arm. Full 200° scans served as the gold standard. From these datasets, reconstructions with reduced orbital rotations (100°–200°, in 20° increments) were generated under three imaging conditions: (1) without a metal implant, (2) with plate osteosynthesis and extra-articular screw configuration, and (3) with plate osteosynthesis and intra-articular screw configuration. Both configurations were reconstructed with and without metal artifact reduction (MAR), resulting in 30 datasets per specimen. Three blinded raters independently assessed subjective image quality, visualization of the articular surface, intra-articular screw detection, and diagnostic certainty.

**Results:**

A reduction of orbital rotation had a significant overall effect on subjective image quality and assessability of articular surfaces (*p* < 0.001). Post-hoc analysis showed no further significant improvement in image quality beyond 160° of orbital rotation. MAR significantly enhanced image quality and surface visualization (*p* < 0.001). Sensitivity in detecting intra-articular screws was preserved down to an orbital rotation of 160°, while specificity was unaffected by rotation reduction (*p* = 0.519). MAR did not significantly influence screw detection accuracy.

**Conclusions:**

Intraoperative 3D imaging with a 160° orbital rotation yields sufficient image quality, enabling the reliable identification of intra-articular screws during plate osteosynthesis of the proximal humerus. The additional use of MAR enhances visualization in the presence of metal without increasing the detection rate of misplaced screws. 3D imaging with reduced orbital rotation minimizes spatial demands and collision risks, supporting its practical application as an effective intraoperative imaging technique for the shoulder.

## Background context

Proximal humerus fractures account for about 5–6% of all adult fractures [1, 2]. These fractures predominantly occur in elderly individuals, with women being affected more frequently [3, 4]. When surgical treatment is required, locking plate osteosynthesis is widely used as the primary method of fixation [5, 6]. This approach, however, is associated with a relatively high complication rate. Meta-analytic data report a 10.5% revision rate for locking plate osteosynthesis, with screw perforation and avascular necrosis of the humeral head being the most common complications [7].

In the surgical treatment of proximal humerus fractures, intraoperative imaging is typically performed using standard 2D fluoroscopy [8]. However, because only a limited number of views can be obtained, conventional 2D fluoroscopy may be insufficient to fully assess the morphology of the humeral head and its complex anatomical relationships [9]. Consequently, intraoperative 3D imaging has been shown to provide superior accuracy and reliability in detecting intra-articular screw penetration compared with conventional fluoroscopy in proximal humeral fracture models [10].

In the clinical setting, implementing intraoperative 3D imaging is more challenging than in experimental models. The anatomical position of the proximal humerus in proximity to the thorax complicates C-arm positioning and limits the feasibility of collision-free 3D image acquisition.

Previous studies using non-isocentric C-arms placed patients in a modified beach-chair position on a carbon fiber table, allowing transverse C-arm positioning from the opposite side [9, 11]. This setup increases radiation exposure to the thorax and its radiosensitive organs, limits optimal shoulder visualization during intraoperative 2D imaging, and requires substantial modifications to the operating room environment.

Böhringer et al. described an alternative technique using an isocentric C-arm in a parasagittal position with the upper extremity fixed on a support arm, which enables both 2D fluoroscopy and 3D imaging without major modifications to the surgical setup [8]. In practice, however, its feasibility can be affected by patient size or variations in operating room environment.

A potential strategy to overcome these limitations is to reduce the amount of orbital rotation during intraoperative 3D imaging with an isocentric C-arm. This approach minimizes the spatial requirements for C-arm movement while enabling the reconstruction of a 3D volume from a limited number of 2D projections, a technique known as tomosynthesis [12]. However, the resulting dataset is inherently incomplete, which may compromise image quality and diagnostic accuracy [13].

This cadaver study systematically evaluated the effect of progressive reduction of orbital rotation during intraoperative 3D imaging on image quality and the reliability of intra-articular screw detection in locking plate osteosynthesis of proximal humerus fractures.

## Methods

### Ethical approval

The study was approved by the ethics committee (application number: 2024–17547; date: May 7, 2024). The cadavers were obtained from Science Care (Science Care, Phoenix, AZ, USA). Consent from the body donors for use in medical research was obtained during the donors’ lifetime. All procedures were in accordance with the ethical standards of the institutional and national research committee and with the 1964 Helsinki declaration and its later amendments or comparable ethical standards.

### Experimental setup

For this study, an isocentric 3D C-arm (Cios Spin, Siemens Healthineers AG, Erlangen, Germany) was used. During a 200° orbital rotation, the system acquires up to 400 images to generate a 3D dataset. The orbital rotation can be flexibly adjusted between 120° and 200° during scan acquisition, allowing for variable image acquisition protocols.

Five fresh-frozen cadaveric shoulder specimens, including scapula and humerus, were used for this study. The specimens were mounted in a 3D-printed clamp on a carbon table in a position corresponding to the intraoperative beach-chair setup. The C-arm was positioned in a parasagittal orientation relative to the specimen. First, a 3D scan with complete 200° orbital rotation (gold standard) was performed without any metal implants. Second, osteosynthesis of the proximal humerus was carried out using a locking plate (PHILOS, DePuy Synthes, Umkirch, Germany). The plate was instrumented according to standard clinical practice, with six screws placed in the humeral head (A- and B-level screws as well as E-level calcar screws) and one to two screws placed in the shaft region.

For each specimen, two screw configurations were created: an extra-articular configuration with screws placed as long as possible without penetrating the articular surface, and an intra-articular configuration with one screw tip intentionally placed just through the articular surface using the shortest possible screw to simulate borderline conditions for detection. Screw malposition was confirmed on the 200° 3D scan and by direct visualization through an anterior arthrotomy.

### Reduction of orbital rotation

**Fig. 1:**
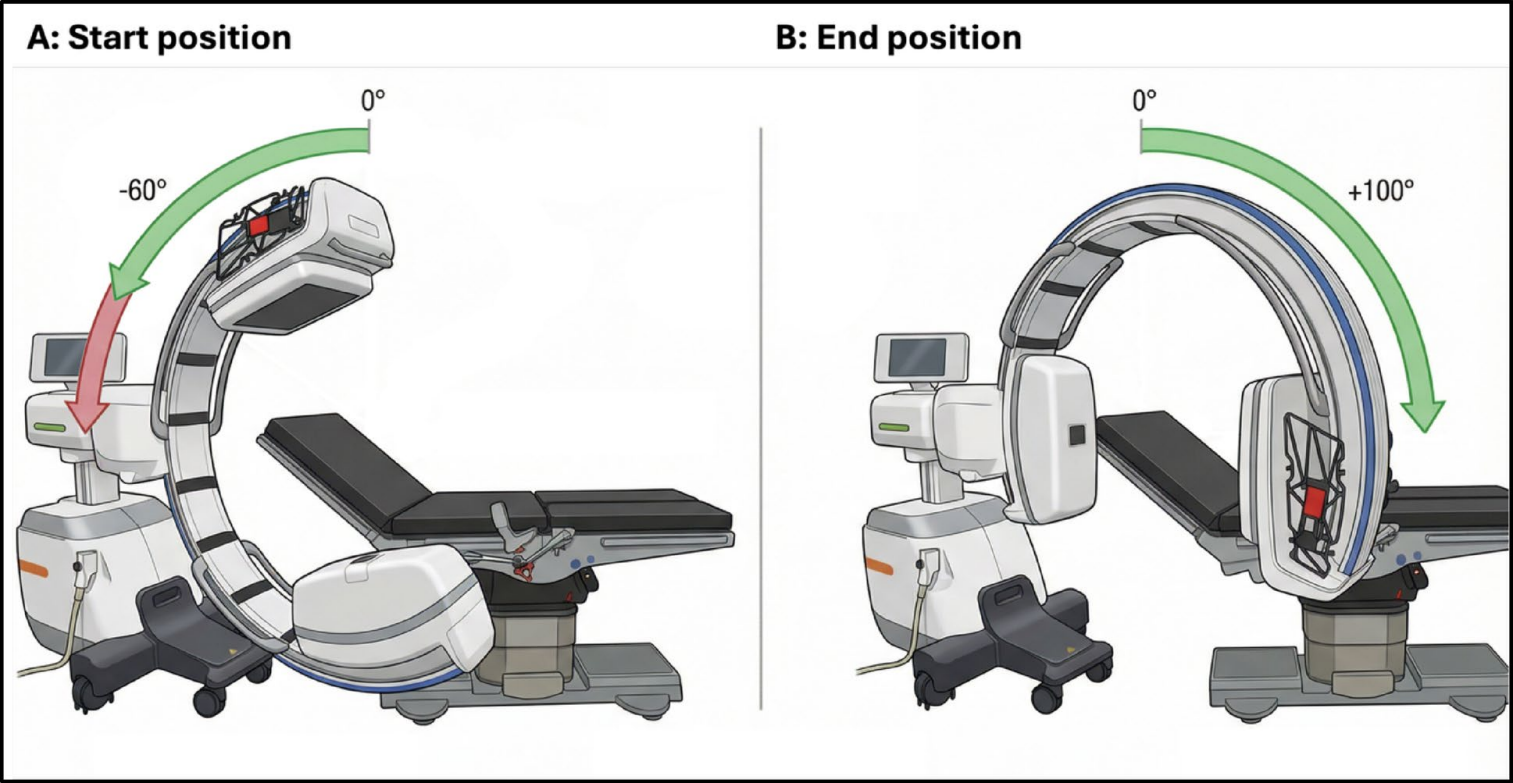
Intraoperatively achievable orbital rotation range.Orbital motion was limited to − 60° in the negative direction (**A**) due to C-arm–table collisions, while + 100° were attainable in the positive direction (**B**), yielding an effective rotation range of − 60° to + 100° (160° total)

To achieve a controlled reduction of orbital rotation, a dedicated manufacturer-provided reconstruction software was used, applying the same reconstruction algorithms as the C-arm’s internal 3D imaging and metal artifact reduction (MAR) pipeline. The reduced scans were calculated retrospectively from the complete 200° datasets by defining specific start and end points, thereby selecting the desired orbital span. All reconstructions were performed both with and without metal artifact reduction (MAR) to assess its additional effect on image quality. In intraoperative positioning tests, orbital rotations ranging from − 60° to 100° (total 160°) were achievable without collisions (Fig. 1). Collisions between the X-ray tube and the operating table frequently limited orbital rotation. Based on these findings, orbital rotation was systematically reduced in 20° increments. An asymmetric reduction from 200° to 160°, and a symmetric reduction from 160° down to 100°, were simulated.

### Assessment of 3D datasets

Three raters (two experienced orthopedic trauma surgery residents and one fifth-year medical student) independently reviewed the 3D datasets in a randomized and blinded manner. Image quality was assessed using the criteria for subjective image assessment described by Stübig et al. [14], as outlined in the questionnaire presented in Table 1. Overall subjective image quality was quantified as the mean score of the first six questions. In addition, screw position was assessed as a separate binary outcome (intra-articular vs. extra-articular), and the raters’ certainty in screw position assessment was rated using the corresponding Likert-scale item of the questionnaire.

**Table 1:** Questionnaire for image quality assessment

Score	Subjective image quality	Cortical bone visualization	Trabecular bone visualization	Assessability of articular surfaces	Artifacts	Overall clinical assessment	Certainty in screw position assessment
1	Reduced	Not visible	Not visible	Not visible	Very disturbing	Not possible	Very low
2	Slightly reduced	Barely visible	Barely visible	Barely visible	Disturbing	Limited	Low
3	Acceptable	Acceptable	Acceptable	Acceptable	Moderate	Possible	Moderate
4	Good	Good	Good	Good	Few	Good	High
5	Very good	Very good	Very good	Very good	None	Very good	Very high

### Statistics

Statistical analyses were performed using IBM SPSS Statistics, Version 29 (IBM Corp., Armonk, NY, USA). Data are presented as mean ± standard deviation (SD), with a significance level of α = 0.05. Although the data were not normally distributed, the mean and standard deviation were reported because the 5-point Likert scale was assumed to have equal intervals. For non-normally distributed paired data with more than two levels, the Friedman test and Dunn–Bonferroni post-hoc tests were applied. Significance levels were adjusted for multiple testing using the Bonferroni correction. For two-level comparisons, the Wilcoxon test was used. A Chi-square test was applied to compare sensitivity and specificity between groups regarding the detection of intra-articular screw penetration. Effect sizes were interpreted according to Cohen [15]. Inter-rater reliability (*n* = 3) was assessed using the intraclass correlation coefficient (ICC) and interpreted according to Cicchetti’s recommendations [16].

## Results

### Interrater reliability

1140 data points were assessed per rater, based on the number of reconstructed datasets and questionnaire items evaluated. The analysis demonstrated excellent inter-rater reliability in the rating of image quality (ICC = 0.927, 95% CI 0.914–0.938). Given the excellent level of agreement, analyses for subjective image quality were conducted using the mean values across all raters.

### Subjective image quality (IQ)

**Fig. 2:**
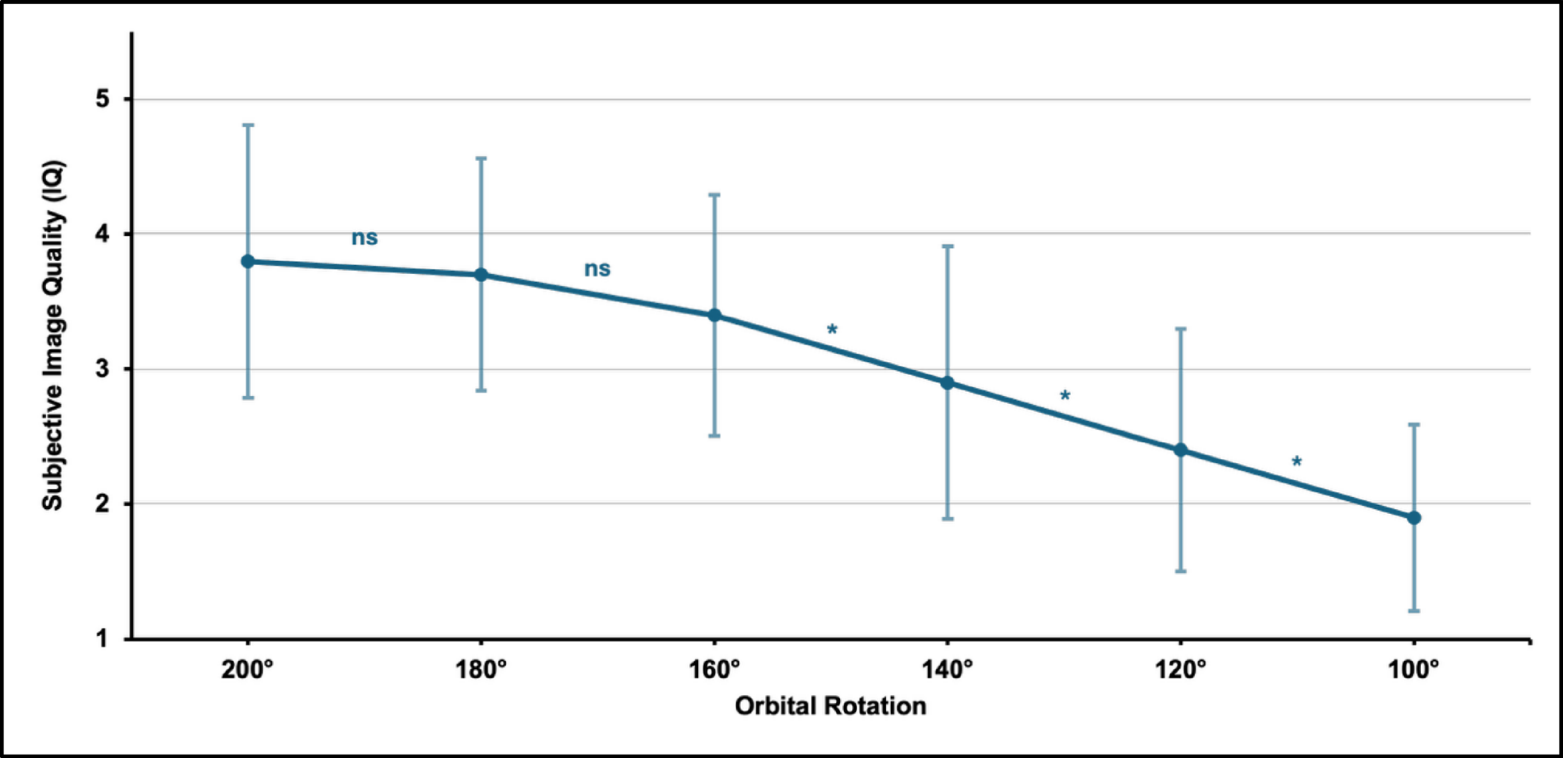
Mean subjective image quality (IQ) scores at different orbital rotations. Asterisks indicate statistically significant differences between adjacent orbital rotation steps in post-hoc analysis (Dunn–Bonferroni; *p* < 0.05; ns = not significant)

Decreasing orbital rotation resulted in a significant reduction of subjective image quality (IQ) when averaged across all reconstructed scans (*p* < 0.001; Fig. 2). Including all scans, there was no further significant improvement in IQ from an orbital rotation of 160° onwards. A medium effect (*r* = 0.318) was found over the entire reduction (100°–200°).

Post-hoc analyses showed a significant difference in orbital rotation between 100° and 160°. Weak effects were observed for each 20° reduction step (*r* = 0.075–0.097).

Based on the predefined Likert-scale interpretation, an IQ score of ≥ 3 was considered acceptable image quality. Using this threshold, acceptable image quality was maintained down to 120° in scans without a metal implant, down to 140° in scans with locking plate osteosynthesis and MAR, whereas scans with locking plate osteosynthesis without MAR reached acceptable image quality only at an orbital rotation of 180°.

**Fig. 3:**
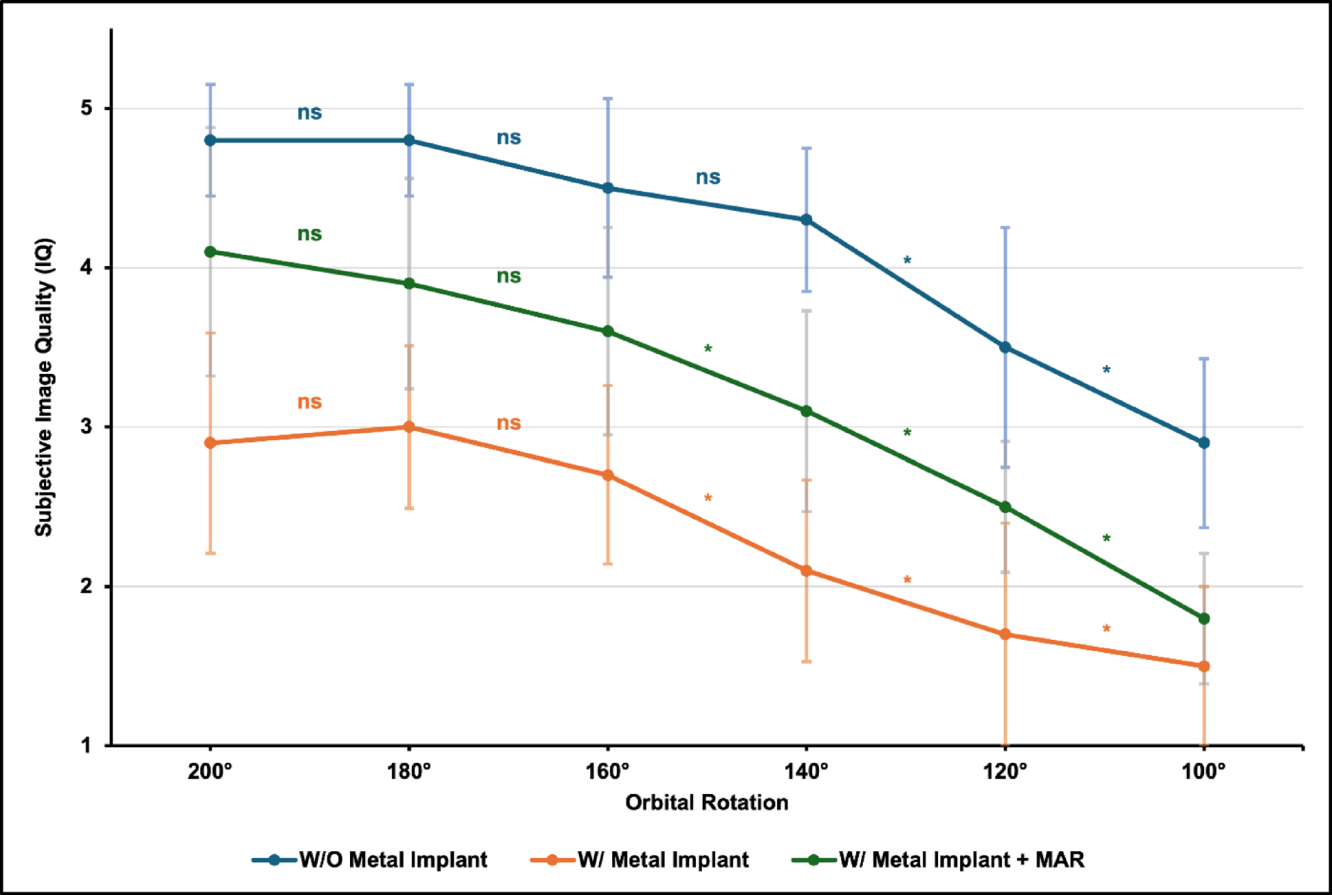
Mean subjective image quality (IQ) scores for scans without a metal implant and with metal implant ± MAR. Asterisks indicate statistically significant differences between adjacent orbital rotation steps in post-hoc analysis (Dunn–Bonferroni; *p* < 0.05; ns = not significant)

Post-hoc analysis demonstrated a statistically significant decline in image quality at lower rotation angles, with significant decreases observed below 140° in scans without metal and below 160° in scans with metal, irrespective of MAR application (Fig. 3).

**Fig. 4:**
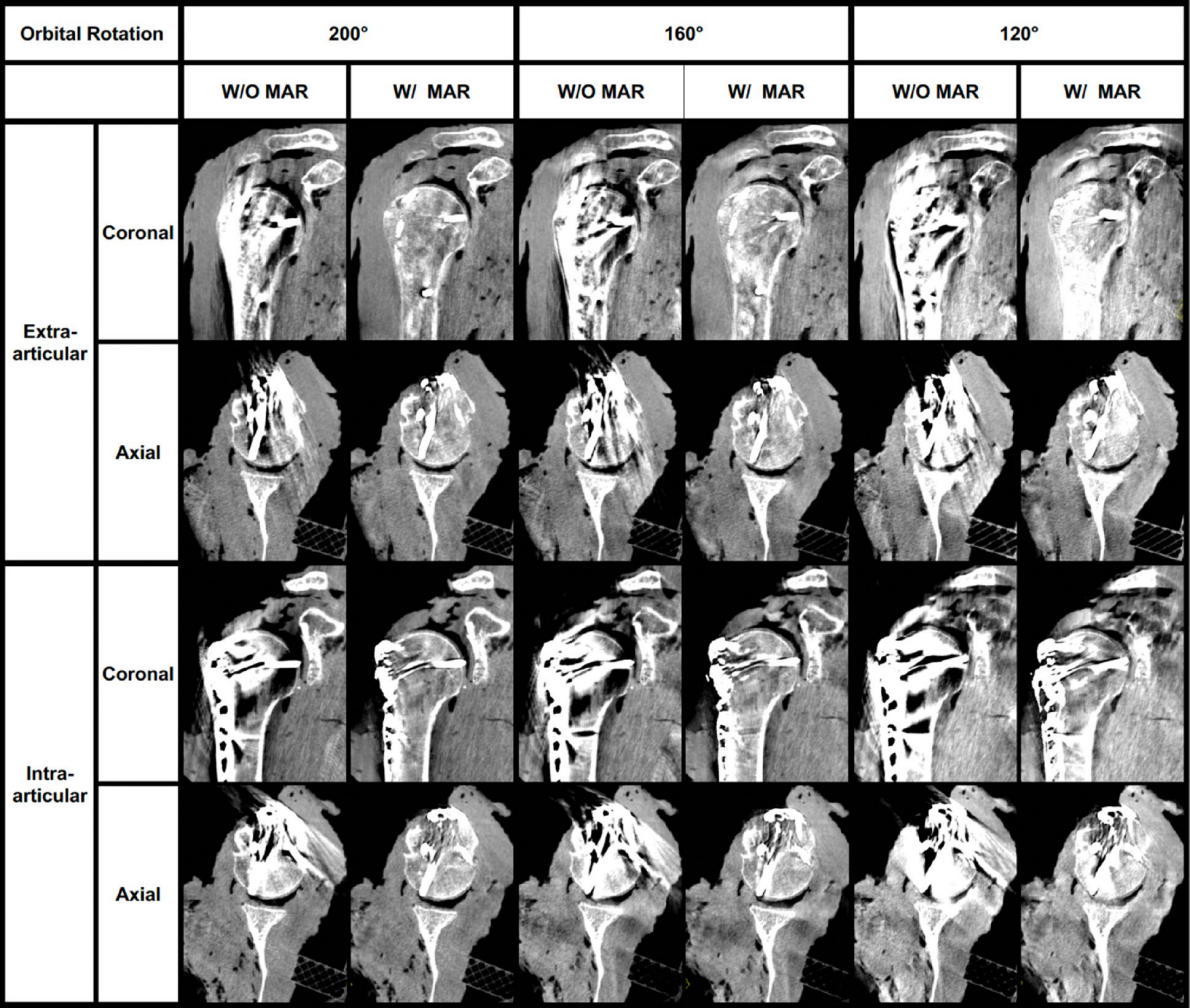
Visual examples in the coronal and axial planes

Post-hoc analysis showed a significant decrease in IQ for rotations below 140° in scans without a metal implant (*p* < 0.001) and for rotations below 160° in scans with locking plate osteosynthesis (*p* < 0.001). Following MAR reconstruction, no further significant improvement in image quality was observed for rotations exceeding 160° (*p* < 0.05). Representative visual examples of image quality and screw assessment across different orbital rotations are shown in Fig. 4.

### Assessability of articular surfaces

**Fig. 5:**
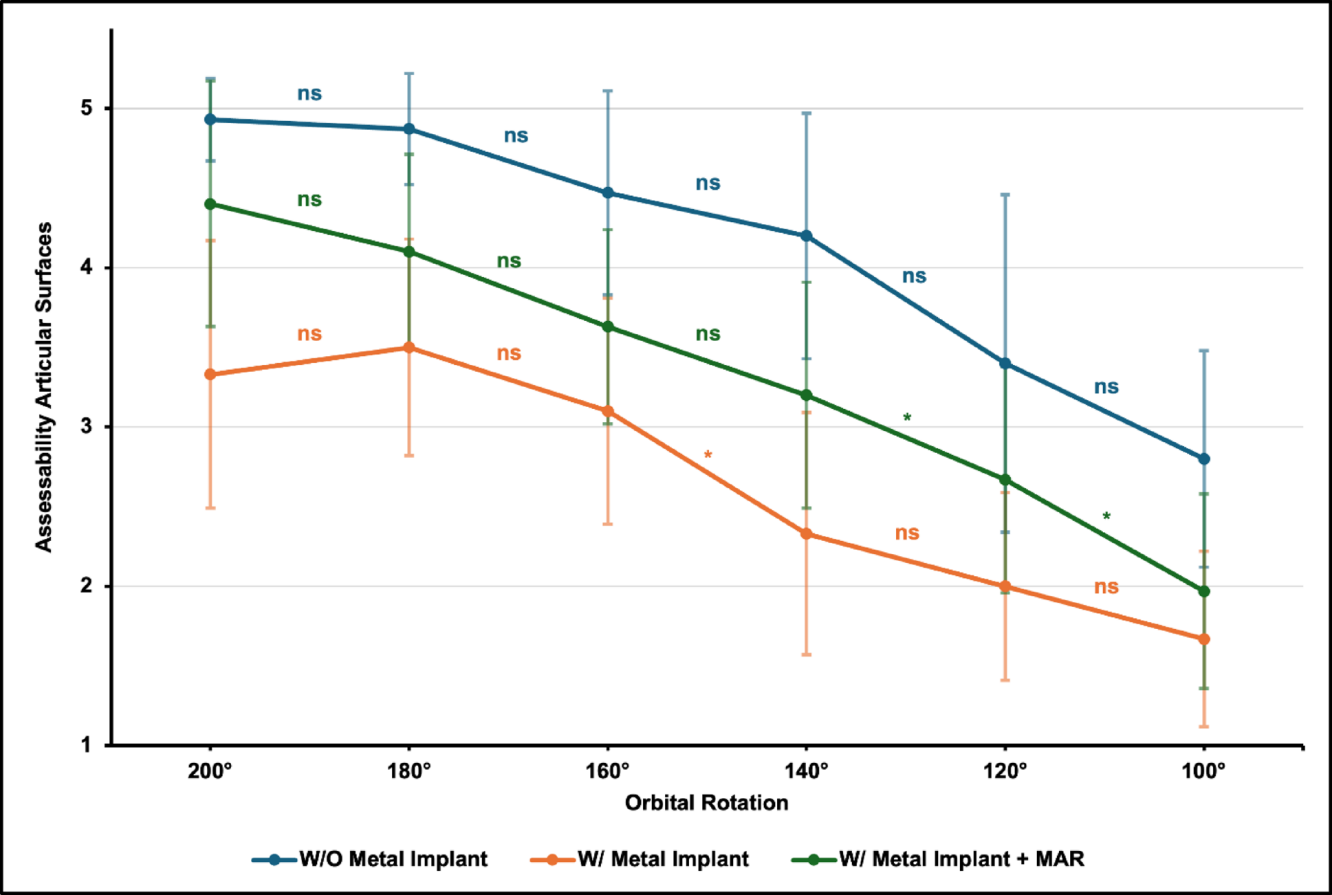
Mean score in “Assessability of articular surfaces” for scans without a metal implant and with metal implant ± MAR. Asterisks indicate statistically significant differences between adjacent orbital rotation steps in post-hoc analysis (Dunn–Bonferroni; *p* < 0.05; ns = not significant)

Overall, assessability of the articular surfaces decreased significantly with progressive reduction of orbital rotation for all subgroups (Fig. 5; all *p* < 0.001). Application of MAR algorithms significantly improved the assessability of the articular surfaces (*p* < 0.001).

Post-hoc pairwise comparisons demonstrated a statistically significant decline in assessability for orbital rotations below 160° in scans with locking plate osteosynthesis without MAR and below 140° in scans with locking plate osteosynthesis with MAR. In scans without a metal implant, no individual rotation step reached statistical significance despite a visible decrease in assessability, which is likely attributable to the smaller number of datasets and conservative correction for multiple testing.

### Detection of intra-articular screws

**Fig. 6:**
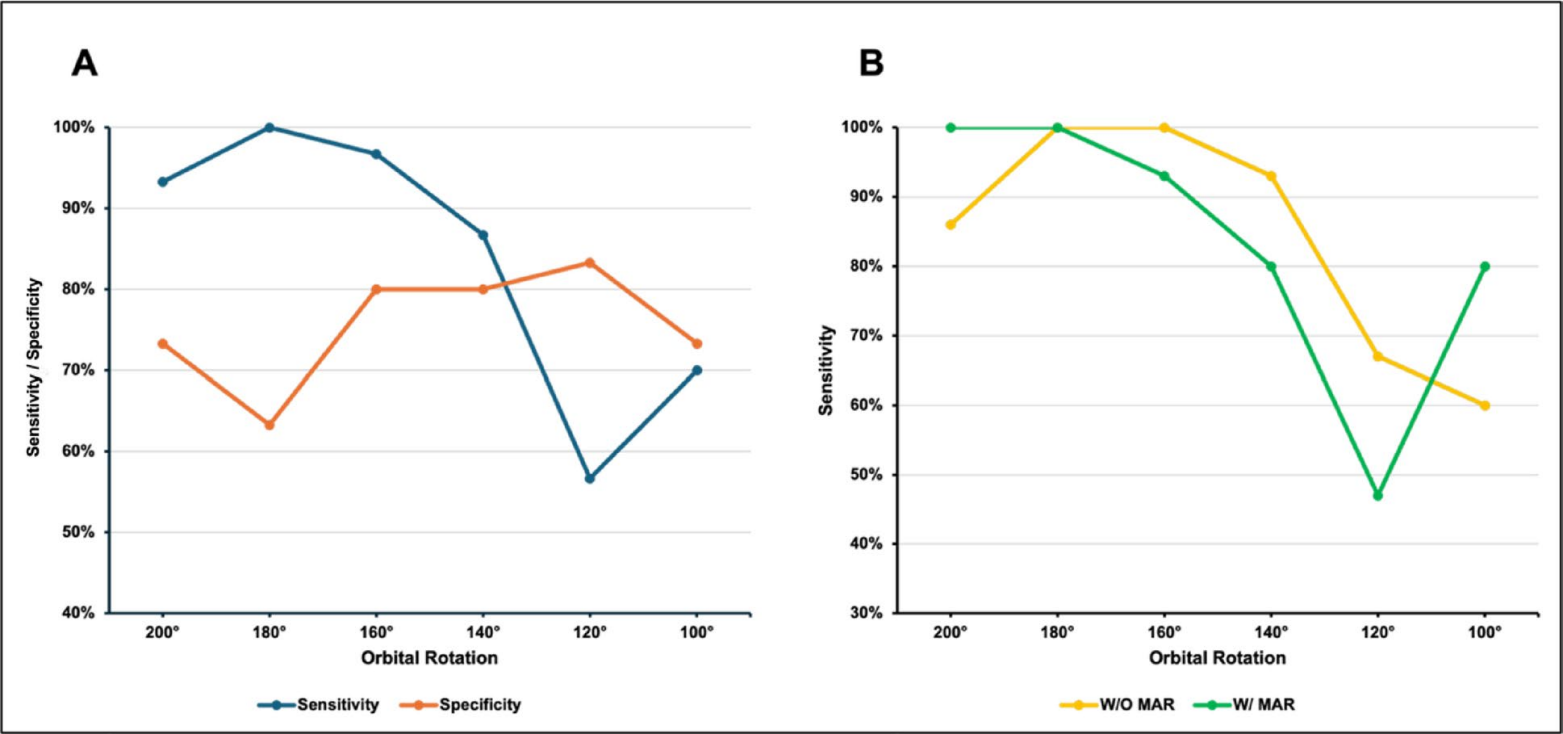
Sensitivity and specificity in the detection of intra-articular screws at different orbital rotations. **A** Global sensitivity and specificity across all datasets **B** Sensitivity with and without MAR reconstruction

Sensitivity in the detection of intra-articular screws decreased significantly with reduced orbital rotation (*p* < 0.001). Specificity for identifying correctly positioned screws, however, was not significantly affected by a reduction in orbital rotation (*p* = 0.519). Figure 6A illustrates the trend of sensitivity and specificity as orbital rotation decreases. Across the entire range, sensitivity remained consistently higher than specificity. The additional application of MAR did not result in a significant improvement in either sensitivity (*p* = 1.0) or specificity (*p* = 0.603) (Fig. 6B).

### Certainty in the assessment of intra-articular screws

Certainty in the assessment of intra-articular screws decreased significantly with reduced orbital rotation (*p* < 0.001). Reconstruction with MAR algorithms significantly increased the certainty in the assessment of intra-articular screw positions (*p* < 0.001). Post-hoc analysis revealed that increasing the orbital rotation beyond 160° did not result in a significant gain in certainty. When using MAR, a decrease in certainty was observed at 140° rotation, and further increasing the orbital rotation beyond 140° provided no significant improvement.

## Discussion

The results of this cadaver study demonstrate that intraoperative 3D imaging of the proximal humerus provides sufficient image quality and enables reliable assessment of screw position, even with reduced orbital rotation. Pairwise comparison with the 200° gold standard showed that rotations down to 160° provided sufficient image quality and assessability when using an isocentric C-arm. The use of MAR algorithms further improved visualization and diagnostic certainty; however, this improvement did not translate into a significant increase in sensitivity for the detection of intra-articular screws.

Conventional intraoperative imaging during locking plate osteosynthesis remains primarily based on 2D fluoroscopy, which provides limited visualization of the complex three-dimensional anatomy of the proximal humerus.

These limitations were clearly demonstrated by Lowe et al., who systematically analyzed the diagnostic accuracy of standard fluoroscopic views for different screw exit locations in cadaveric humeri [17]. They found that while the specificity of fluoroscopic imaging was 100%, meaning that screws appearing intraosseous were truly within bone, the sensitivity averaged only 55% and varied greatly depending on screw location and C-arm orientation.

When comparing these findings, the lower specificity observed in the present study should be interpreted in the context of both the experimental design and the imaging modality. While conventional fluoroscopy demonstrated high specificity for clearly intraosseous screws, intraoperative 3D imaging was deliberately applied to borderline screw configurations placed close to the articular surface. The additional depth information provided by 3D imaging may lead to a more cautious classification of screw position, resulting in an increased number of false-positive findings. From a clinical perspective, this trade-off may be acceptable, as avoiding undetected intra-articular screw penetration remains the primary intraoperative objective.

In this context, intraoperative 3D imaging enables a more comprehensive assessment of implant placement and screw position, allowing immediate correction of suspected malpositions that might otherwise remain undetected.

Theopold et al. evaluated intraoperative 3D imaging using a non-isocentric C-arm system and reported intra-articular or near-articular screw perforations in 18% of cases, leading to immediate intraoperative corrections [11]. Böhringer et al. subsequently demonstrated the feasibility of isocentric 3D imaging in a prospective series, enabling reliable assessment of fracture reduction, implant position, and joint articulation with intraoperative correction of detected malpositions [8]. These studies established intraoperative 3D imaging as a valuable adjunct to conventional fluoroscopy.

In elderly patients with osteoporotic bone, the relevance of reliable intraoperative screw assessment becomes particularly evident. Reduced tactile feedback in cancellous bone impairs accurate determination of screw length, increasing the risk of intra-articular perforation [18, 19]. In this context, intraoperative 3D imaging offers a clear diagnostic advantage by directly visualizing the screw tip relative to the articular surface and enabling correction before wound closure. Furthermore, advanced 3D imaging may reduce reliance on prolonged fluoroscopic sequences and postoperative CT scans, thereby optimizing intraoperative workflow and radiation exposure for the OR team [20].

While previous investigations demonstrated feasibility using full rotational scans, the present cadaveric study specifically addressed the technical challenge of restricted C-arm mobility in shoulder surgery. Our findings indicate that diagnostic image quality, articular surface assessability, and screw detection remain reliable even with reduced orbital rotations. Sensitivity for intra-articular screw detection remained high up to 160°, supporting the clinical feasibility of reduced-angle intraoperative tomosynthesis.

An important observation of the present study is that metal artifact reduction (MAR) did not lead to an improvement in sensitivity for the detection of intra-articular screw positions, despite its beneficial effects on overall image quality and assessability of the articular surface. This finding highlights a relevant discrepancy between subjective image quality improvement and diagnostic performance.

This discrepancy may be explained by MAR-related alterations of metallic structures, resulting in an over-correction of metal artifacts and a potential shortening effect of the visualized screw tips. Such algorithm-induced modifications can reduce the conspicuity of the screw tip and may mask minimal intra-articular perforations, particularly in borderline configurations close to the subchondral bone [21].

From a clinical standpoint, this observation suggests that although MAR reconstruction improves overall image quality and visualization of osseous structures, it does not necessarily enhance diagnostic accuracy for screw position. Therefore, assessment of screw length and joint penetration, especially in borderline cases, should be interpreted with caution and, where possible, considered in conjunction with non-MAR reconstructions and the overall intraoperative imaging context.

This cadaveric study focused exclusively on intraoperative 3D imaging using an isocentric C-arm, without direct comparison to conventional 2D fluoroscopy. While the data allow for an internal comparison to the 200° gold standard, a prospective clinical study would be required to confirm the diagnostic advantages of 3D imaging with reduced orbital rotation over standard intraoperative fluoroscopy in vivo.

In addition to these methodological considerations, this study was conducted using a single isocentric 3D C-arm system. Differences between imaging platforms, including isocentric versus non-isocentric designs, system-dependent reconstruction pipelines, and metal artifact reduction (MAR) algorithms, may influence image quality and 3D dataset generation. Although the general concept of reduced orbital rotation is likely applicable to other isocentric C-arm systems, system-specific technical characteristics may influence the transferability of the findings.

Moreover, the specimens used were non-fractured and therefore did not account for variations in fracture morphology, soft-tissue conditions, or intraoperative image artifacts caused by blood or irrigation fluid. Although screw malposition was simulated to represent borderline intra-articular configurations, articular step-offs and fracture reductions were not modeled, limiting conclusions regarding postoperative joint congruence. Finally, the sample size was limited, which may reduce statistical power and limit the detection of subtle differences between orbital rotation angles, and subjective image assessment may have been influenced by the raters’ experience level despite high interrater reliability.

### Conclusion

Intraoperative 3D imaging with a reduced orbital rotation of 160° provides sufficient image quality and reliable identification of intra-articular screws during locking plate osteosynthesis of the proximal humerus. The additional use of MAR algorithms significantly enhances image quality in the presence of metallic implants, without increasing the detection rate of misplaced screws. This approach, with reduced orbital rotation, offers a practical intraoperative imaging option that maintains diagnostic accuracy while minimizing spatial demands. These results support the broader implementation of intraoperative 3D imaging as a valuable tool for improving implant assessment.

## Data Availability

The datasets generated during and/or analyzed during the current study are available from the corresponding author on reasonable request.

## Declarations

The cadavers were obtained from Science Care (Science Care, Phoenix, AZ). Consent from the body donors for use in medical research was obtained during the donor’s lifetime.
